# Strategic purchasing for universal health coverage: examining the purchaser–provider relationship within a social health insurance scheme in Nigeria

**DOI:** 10.1136/bmjgh-2018-000917

**Published:** 2018-10-25

**Authors:** Enyi Etiaba, Obinna Onwujekwe, Ayako Honda, Ogochukwu Ibe, Benjamin Uzochukwu, Kara Hanson

**Affiliations:** 1 Health Policy Research Group, Department of Pharmacology and Therapeutics, College of Medicine, University of Nigeria, Enugu, Nigeria; 2 Department of Health Administration and Management, College of Medicine, University of Nigeria, Enugu, Nigeria; 3 Department of Economics, Sophia University, Tokyo, Japan; 4 Department of Community Medicine, College of Medicine, University of Nigeria, Enugu, Nigeria; 5 Department of Global Health and Development, London School of Hygiene and Tropical Medicine, Keppel Street, London, UK

**Keywords:** strategic purchasing, purchaser provider split, contracts, provider payment methods, National Health Insurance Scheme, Formal Sector Social Health Insurance Programme, Nigeria

## Abstract

**Background:**

In an attempt to achieve universal health coverage, Nigeria introduced a number of health insurance schemes. One of them, the Formal Sector Social Health Insurance Programme (FSSHIP), was launched in 2005 to provide health cover to federal government and formal private sector employees. It operates with two levels of purchasers, the National Health Insurance Scheme (NHIS) and health maintenance organisations (HMOs). This study critically assesses purchasing arrangements between NHIS, HMOs and healthcare providers and determines how the arrangements function from a strategic purchasing perspective within the FSSHIP.

**Methods:**

A qualitative study undertaken in Enugu state, Nigeria, data were gathered through reviews of documents, 17 in-depth interviews (IDIs) with NHIS, HMOs and healthcare providers and two focus group discussions (FGDs) with FSSHIP enrolees. A strategic purchasing lens was used to guide data analysis.

**Results:**

The purchasing function was not being used strategically to influence provider behaviour and improve efficiency and quality in healthcare service delivery. For the purchaser–provider relationship, these actions are: accreditation of healthcare providers; monitoring of HMOs and healthcare providers and use of appropriate provider payment mechanisms for healthcare services at every level. The government lacks resources and political will to perform their stewardship role while provider dissatisfaction with payments and reimbursements adversely affected service provision to enrolled members. Underlying this inability to purchase, health services strategically is the two-tiered purchasing mechanism wherein NHIS is not adequately exercising its stewardship role to monitor and guide HMOs to fulfil their roles and responsibilities as purchasing administrators.

**Conclusions:**

Purchasing under the FSSHIP is more passive than strategic. Governance framework requires strengthening and clarity for optimal implementation so as to ensure that both levels of purchasers undertake strategic purchasing actions. Additional strengthening of NHIS is needed for it to have capacity to play its stewardship role in the FSSHIP.

**Key questions**

**What is already known?**

- The Formal Sector Social Health Insurance Programme (FSSHIP) is a social health insurance programme in Nigeria, run by the National Health Insurance Scheme (NHIS).
- Services are purchased for the NHIS by health maintenance organisations (HMOs) from both public and private healthcare providers; but little is known about the effectiveness of these purchasing arrangements in securing efficient and high quality care for members.

**What are the new findings?**

- Because of the weakness in the governance of purchasing arrangements, purchasing under the FSSHIP was mostly passive and not strategic, thus leading to suboptimal service delivery.
- There are conflicts of interests between the two levels of purchasers (ie, NHIS and HMOs)—NHIS (the purchaser and regulator of FSSHIP), has not provided strong leadership over HMOs (the other level of purchaser/purchasing administrator), thus undermining the strategic use of purchasing arrangements with healthcare providers

**What do the new findings imply?**

- Purchasing organisations need to have governance arrangements that promote strategic purchasing—and to achieve this, the capacity of public purchasers and regulators need to be strengthened.

## Introduction

The purchasing of healthcare services involves three sets of decisions: identifying benefit entitlements; selecting healthcare service providers and determining how healthcare services will be purchased, including provider payment mechanisms and contractual arrangements.^1^ In strategic purchasing, purchasers link these decisions to provider behaviour to encourage providers to pursue equity, efficiency and quality in service delivery.^1^

Strategic purchasing is currently receiving renewed and increasing attention as one of the strategies to achieve universal health coverage, and policy debates are moving away from performance-based financing and its reported challenges to a broader approach of strategic purchasing and attention to the wider environment of purchaser–provider relationships.^2–6^ Contracts describe how resources are transferred from purchasers to providers, how purchasers monitor providers, how providers are accountable to purchasers for their performance and how purchasers make decisions and are accountable to providers for those decisions.^7 8^ The government is expected to play a stewardship role in the purchasing arrangement, fitting this arrangement into a nation’s health policy framework.^2^

In strategic purchasing, purchasers use pooled funds to buy healthcare services for certain groups or the entire population, using levers that encourage healthcare providers to improve health service quality and efficiency. In addition, as the purchaser represents the people in purchasing decisions, they must ensure that mechanisms are in place to identify and reflect people’s needs, preferences and values in purchasing decisions, and hold healthcare providers accountable to the people. Furthermore, government is required to play a stewardship role by providing a clear policy framework and appropriate guidance to ensure that resource allocation and purchasing decisions are linked to public health priorities.^9^

Strategic purchasing entails some form of contract between purchasers and providers and encourages the organisational separation of purchasers and providers in order to facilitate the contractual relationship.^7^ To strengthen the purchasing function of healthcare financing and move towards strategic purchasing, many Asian and African countries have been undertaking healthcare purchasing reform and a number of countries have introduced, or are planning to introduce, mandatory insurance mechanisms. This approach facilitates the organisational split between purchasers and providers.^2^ While empirical studies on healthcare purchasing in low-income and middle-income countries (LMICs) setting are limited in number, recent studies show that purchasing is still predominantly passive and a number of factors constrain strategic purchasing including: fragmentation of pooled funds; beneficiary dissatisfaction with benefit packages and quality of service and provider payment challenges.^10–14^

Nigeria has seen the development of a service delivery model in which third party payers are organisationally separate from service providers, and the operations of the providers are managed by contracts,^15^ also known as a ‘purchaser–provider split’. This model is used within the Formal Sector Social Health Insurance Programme (FSSHIP) that was established in 2005 under the National Health Insurance Scheme (NHIS) to provide formal sector employees (both public and private) with financial risk protection when accessing healthcare services. Under the FSSHIP, the NHIS, a public purchaser, contracts health maintenance organisations (HMOs) as mid-level purchasers to manage contracts with public and private healthcare providers.^16^ The FSSHIP stipulates that employers contribute 10% and employees 5% of their basic salaries. It is a federal government scheme and states are not legally obliged to join the programme. All federal public sector employees and their dependants (spouse and four children under 18 years) automatically qualify for enrolment in the programme. FSSHIP uses HMOs as purchasers. HMOs can be privately or publicly owned and are accredited and registered by the NHIS to purchase healthcare services from providers on behalf of the NHIS.^17^ HMOs do not directly provide healthcare services and are not allowed to do so.^18^ HMOs can function at the national, zonal or state levels. National HMOs should, ideally, have an office in each of Nigeria’s 36 states; zonal HMOs should have an office in every state of the relevant geopolitical zone that they represent; and state HMOs, in addition to a state head office, should have offices in every senatorial district in the state (three per state) and in every local government area where there are up to 5000 enrolees.^17^ The national, zonal and state HMOs perform the same functions and differ only in terms of the share capital requirement to be remitted to NHIS and the geographical areas they cover.^17^

Providers are a mix of public and private healthcare facilities. NHIS requires that they offer beneficiaries the services outlined in predetermined benefit packages, comply with an essential drug list and act as gatekeepers for NHIS in relation to referrals.^17^ Providers receive capitation payments for primary healthcare services and fee-for-service (FFS) payments for hospital care from NHIS, through HMOs, when FSSHIP members access healthcare services. Beneficiaries are allocated to HMOs by NHIS. If beneficiaries are not satisfied with services, they can change providers after a given period of time but are unable to change HMOs.^17^ Table 1 below summarises what is purchased, for whom and by whom.

**Table 1:** A description of the Nigerian FSSHIP

Purchasers	NHIS and HMOs. NHIS purchases tertiary services directly from state and federal tertiary care providers while primary and secondary care services are purchased through HMOs. They receive funds from the national government through the NHIS to purchase healthcare packages.
What services are purchased?	Predetermined primary, secondary and tertiary care packages with some partial and total exclusions. Packages are same for all enrolees and are decided by the NHIS.
Who uses the services?	Federal civil servants and organised private sector (formal private sector organisations with more than 10 employees). Coverage is only about 5% of the Nigerian population.
Who provides services?	A mix of public, private and faith-based organisations.
How are providers paid?	Primary care health services are reimbursed through capitation, secondary and tertiary care through fee-for-service (FFS). Public and private providers are reimbursed at the same rate.

FSSHIP, Formal Sector Social Health Insurance Programme; HMOs, health maintenance organisations; NHIS, national health insurance scheme.

This paper critically examines how the contractual arrangements between healthcare providers and the two-tiers of FSSHIP purchasers function from a strategic purchasing perspective. It also provides information on how the flow of resources, information (including monitoring and communication) and decision-making authority shape the purchaser–provider relationship.

## Methods

This study was undertaken in Enugu state, Nigeria. Enugu state, one of 36 states in Nigeria, is situated in the southeast geopolitical zone. Since the FSSHIP currently covers only federal employees, it is felt that findings from Enugu state are likely to be generalisable to other states, limited only by local contextual factors. The population of Enugu state was estimated to be approximately 3.9 million in 2013.^19^ Table 2 below highlights the contextual characteristics of Enugu state in relation to the FSSHIP.

**Table 2:** Contextual characteristics of Enugu state, Nigeria

Variable	Finding
Population estimate in study period (2013)	3.9 million
Federal government establishments	87
Number of federal employees	18 000 (0.46% of the population)
NHIS registered providers at time of study	70
Registered HMOs at time of study	16
Total number health facilities	962
Public facilities	4 tertiary, 52 secondary, 492 primary health facilities
Private facilities	96 secondary, 242 primary health facilities

Source.^19 34^

HMOs, health maintenance organisations; NHIS, national health insurance scheme.

### Data collection

Data was collected by review of documents, in-depth interviews (IDIs) and focus group discussions (FGDs). A total of 17 IDIs (4 NHIS purchasers, 6 HMO purchasers, 4 public providers and 3 private providers) and 2 FGDs were conducted (1 with 8 male FSSHIP members and 1 with 12 female FSSHIP members). Documents were primarily used to establish the formal, de jure relationship as set out in policy/programme guidelines while interviews and FGDs and IDIs were then employed to examine what happens in practice.

Participants in IDIs and FGDs were chosen to reflect the four main actor groups involved in healthcare purchasing—purchasers, providers, citizens and government—as identified using the analytical framework explained below. IDIs and FGDs were used to elicit relevant and in-depth information for each of the actor groups involved in healthcare purchasing and triangulate the data obtained in the study.

IDIs were undertaken with: Enugu state NHIS officials (in both purchasers and government roles), HMOs as healthcare purchasing administrators and healthcare providers, both public and private. An initial list of potential healthcare providers was subject to purposive selection that considered: the level of healthcare services (primary, secondary and tertiary) provided, a mix of public and private providers. Those providers who offered more than one level of service were identified as key respondents. Interviews was discontinued until no new information was obtained (saturation). To obtain HMO participants for the IDIs, 16 HMOs were identified as having offices in Enugu state, of these six gave consent to be included in the study.

Two FGDs were held with FSSHIP members, one with male FSSHIP members and another with female members. Participants in the FGDs were purposively sampled from six (6) Federal Ministry offices in Enugu state (the target population of FSSHIP was originally federal government workers, but the NHIS is currently trying to expand coverage). Administrative officers assisted in the selection of two participants from each Ministry. Where they exist, the health insurance desk officer was included in the FGD due to their role linking the insurance scheme and Ministry staff.

Interviews and discussions were conducted in English by trained interviewers and facilitators; audio-recorded with study participants’ consent and transcribed verbatim. The accuracy of transcriptions was checked against audio-recordings by the IDI interviewers and FGD facilitators before coding was undertaken.

### Analytical framework

This study was undertaken as part of a multicountry study that examined how healthcare purchasing functions in 10 African and Asian countries.^20^ The study applied an analytical framework based on Figueras, *et al*,^21^ to examine key purchasing relationships including: (i) purchasers and providers, (ii) government and purchasers and (iii) citizens and purchasers. The framework was used to develop a list of strategic purchasing actions for each actor group, which then formed the analytical basis for the multicountry study. The analytical framework used in the multicountry study has been reported elsewhere.^22–24^

This study applied the framework to map the actors involved in FSSHIP healthcare purchasing with a focus on identifying the roles and responsibilities of the actors in the purchaser–provider relationship. In strategic purchasing, the purchaser–provider relationship concerns purchasers’ use of financial, contractual, regulatory and monitoring mechanisms as levers to ensure that healthcare providers deliver technically efficient and quality services. The strategic relationship requires purchasers to consider: (1) the criteria used to select providers; (2) the form of contract employed; (3) the mechanisms by which providers are paid; (4) how prices for services are set, and whether prices are affordable and realistic and (5) the mechanisms put in place to monitor performance. The analytical framework, based on ‘ideal’ purchasing actions, was used to identify how purchasers: (1) select providers, considering the range and quality of services and location of providers; (2) establish service agreements/contracts; (3) develop formularies and standard treatment guidelines; (4) design provider payment methods and establish payment rates that encourage efficiency and service quality; (5) audit provider claims; (6) monitor provider performance and act on poor performance; (7) make timely payments to providers and (8) allocate resources equitably between providers.^20^

These ideal strategic purchasing actions were compared with what is expected from purchasers as indicated in policy, law and regulations (the policy design) to identify policy design gaps; and the policy design was compared with what purchasers actually do in current purchasing practices (actual practice) to identify implementation gaps. The purchasing relationship framework and the list of strategic purchasing actions informed the creation of initial codes for data analysis.

### Data analysis

Data were analysed using QSR NVIVO 10 software. Thematic analysis was undertaken using the study’s analytical framework. Analysis started with familiarisation by reading and re-reading the datasets. A list of themes built around the purchasing actions was generated from the framework and formed the initial set of codes for analysis. Additional codes were added to this list to accommodate key issues that emanated from the dataset. The comprehensive list of codes was then used to analyse the whole data set.

## Findings

Findings first describe how NHIS selects, contracts and regulates HMOs and how both the NHIS and HMOs share responsibility for the purchase of healthcare services for FSSHIP members. Subsequently, findings are presented on the selection of healthcare providers, monitoring of healthcare provider performance by purchasers, provider payment mechanisms and inclusiveness of the purchasing decision-making process.

### Selection and regulation of HMOs

NHIS accredits, reaccredits, registers and contracts HMOs to play an intermediary (or purchasing administrator) role between NHIS and healthcare providers. To achieve accreditation, HMOs must first apply for a specified license (ie, state, zonal or national HMO). NHIS then visits the HMO and checks that the HMO is (i) registered as a limited liability company, (ii) registered with the NHIS, (iii) meets the required minimum capital base and (iv) has the basic infrastructural requirements to operate as a national, zonal or state HMO, as stated in the Revised NHIS ACT.^18^ Specifically, HMOs manage contracts between NHIS and healthcare providers for the provision of primary and secondary healthcare services. The contract outlines the conditions of service delivery, including payment methods, arbitration processes, the duration of the agreement and conditions for termination or amendment of the agreement. HMOs are not involved in the selection or accreditation of healthcare facilities but are required to work with providers accredited by the NHIS. HMOs are expected to establish a tripartite agreement with accredited facilities (which is co-signed by NHIS) and the NHIS.^17^ NHIS in turn regulates the HMOs and providers. NHIS pays HMOs an administration fee for their services, which is 10% of the payment to the providers. Because they act primarily as financial intermediaries in the relationship between the NHIS and the providers, HMOs bear no financial risk.^25^

Findings revealed that, in practice, the NHIS seldom undertakes the reaccreditation process or the periodic review of either healthcare providers or HMOs, partly due to financial and human capacity constraints. There have been cases where sanctions have been imposed on HMOs following reviews, but these cases are rare and not all HMOs and facilities are reviewed within a given timeline.

In a year we were supposed to carry out monitoring and accreditation of about three thousand facilities per zone. You’d find out that you can’t go to some facilities even once……NHIS wants to be regulating the private health insurance. They want also to be regulating social health insurances [including HMOs] and they also want to be dictating the quality assurance and they don’t have the means (IDI with NHIS staff member 1).

Many HMOs are owned by financially and politically affluent citizens, some of who serve as members of the NHIS governing council as there is currently no legislation prohibiting this. This appears to constrain the ability of NHIS to effectively regulate HMOs.

Many of the big shots in the country have their HMOs. How do you want to tell them that their HMO is not functioning? You can’t say that. They will ask you why you think you are there, and they will remove you…in fact, at a time the chairman of our NHIS, appointed by the president, was an HMO owner*…* (IDI with a NHIS staff member 1)

*…*when you want to take action [to regulate HMOs], they [HMOs] will move in on the ES [executive secretary] and lobby him, the ES will tell you to please leave them [HMOs]. So, you won’t have anything sufficient when you [NHIS as a regulator or a top layer purchaser of FSSHIP] want to move against them [HMOs] and that is that*…* (IDI with a NHIS staff member 4)

### Selection of healthcare providers—the accreditation mechanism

NHIS accredits and registers healthcare providers and is supposed to reaccredit them after a probation period of 2 years. Providers are selected for accreditation by the NHIS based on their ability to provide primary, secondary or tertiary care services. They are expected to satisfy a set of minimum requirements that includes facility level requirements, personnel requirements (both scope and skill), equipment and registration requirements with relevant professional bodies. These requirements are tailored to each type of provider, that is, primary, secondary or tertiary.^17^ The NHIS also decides, following inspection, whether a facility will offer primary or secondary care or both to FSSHIP enrolees.

As mentioned above, in practice, NHIS seldom reaccredits healthcare providers after the probationary period due to a lack of human and financial resources and a relatively large number of healthcare providers requiring reaccreditation. Consequently, healthcare providers who initially received accreditation from NHIS often continue providing healthcare services to enrolees under NHIS without reaccreditation.

Some providers nominate themselves for accreditation and initiate the accreditation process. Self-nomination can be seen as positive willingness to participate in the scheme and to prepare for accreditation and can draw the attention of NHIS to a facility sooner than if the facility had waited for a routine NHIS provider identification and inspection.

…they [healthcare providers] apply that they want to be accredited with the NHIS. But it’s just an application; it could be obliged that opportunity or denied after they’ve come for inspection. (IDI with a HMO staff member 2)

### Monitoring and accountability mechanisms

HMOs are required to make quarterly visits to healthcare providers to ensure quality and efficiency in healthcare service provision. As stipulated in Section 44 of the revised NHIS Act, healthcare providers must institute programmes that ensure: (i) healthcare services are of good quality and high standard; (ii) basic healthcare services are of uniform standard throughout the country; (iii) the use of medical technology and equipment is consistent with actual needs and standards of medical practice; (iv) medical procedures and the administration of drugs are appropriate and comply with accepted medical practice and ethics and (v) the medication used in the provision of healthcare in the country is included on the Essential Drug List published by the Federal Ministry of Health.^18^ Healthcare providers are also required to send information on health service outputs (an ‘encounter form’) for individual patients they see, to NHIS through HMOs. HMOs are to develop guidelines on how to carry out these functions.

Most HMOs are aware they are required to pay quarterly monitoring visits to registered providers; but they have not developed and harmonised clear guidelines for these visits and there seems to be large variations in the frequency and approach to monitoring visits to providers by HMOs.

Yes, they come in and try to interview the enrolees. Maybe they can come into the hospital and see patients around. In that way, they start asking them what challenges they face. Are you getting the services you want? In that way they monitor service utilisation. (IDI with a public provider 7)

Seldom, they do come; just seldom. But sometimes, they organise what you call providers forum that they have an association they call HEMCAN, that is, Health Maintenance Organisation Association of Nigeria. Then, they usually call up all the providers into a forum. It’s usually quarterly, but they don’t keep to it. In the last six months, I don’t think they’ve done much. (IDI with a private provider 5)

Well, I have never seen them; unless they come in my absence*…*I believe that the HMOs should occasionally send their staff down to the facilities where they send patients; at least to interact with the providers to know what their challenges are*…* (IDI with a private provider 4)

Providers report that the monitoring undertaken by HMOs seems to emphasise financial flows rather than healthcare service delivery by providers. Providers base this opinion on the fact that HMOs frequently visit the accounting departments of the healthcare providers.

The Abuja people [the HMO in Abuja office], they normally come [for a financial audit] to the zonal offices four times a year; they will get feedback from us, most of the time, they make use of this hospital as a pilot study to check the performance of NHIS. Then the zonal office, they always come in weekly or twice weekly, because if there is any case that we cannot handle [in terms of the costs of treating the FSSHIP members], we normally refer the matter to them. So that is it, the communication is almost on daily basis with the zonal office.(IDI with an administrative staff at public provider)

In terms of monitoring healthcare service quality, many HMOs make ad-hoc visits to healthcare providers and directly interact with FSSHIP members to assess members’ perception of healthcare service quality (rather than developing a set of indicators to monitor healthcare service delivery). While some HMOs feel that they can rely on their members to provide accurate feedback on provider performance, others are more cautious about relying solely on feedback from members.

*…*you cannot ask a provider whether he is giving quality care and he will tell you no?; he will always admit that he’s giving quality care. So how do you find out? It’s from the patients (IDI with a HMO staff member 4)

That’s the only way…if you get the complaints from the enrolees, some of them can exaggerate. They will tell you what is not there. But when you get down to your own investigation, you will find out that actually, he or she was wrong. So when we do our own quality assurance, we can attest to that. (IDI with a HMO staff member 2)

On the other hand, some providers consider current HMO monitoring systems to be poor and think that there needs to be more interaction with providers to monitor and supervise healthcare service provision.

*…*they (HMOs) should actually have people on ground. You will come, go through the case notes, see what this person [patient] has been using, discuss with the doctor, because he [patient] is your client. He [patient] needs satisfaction, they [HMO] need to come and monitor. They are not interested, all they are interested is in: send bill and we will do…they are not into the practice, they are not into the supervision, but they are supposed to supervise. (IDI with a private provider 5)

*﻿*They should visit the facilities and see what is on ground, you know, and interact closely with the providers so that they can appreciate the problem. Like for the other HMOs, for instance, the ones set up by banks, you know, we don’t see them. It’s only we send bills, they talk to us on phones and all that; so it’s like a faceless communication (IDI with a private provider 4)

### Provider payment mechanisms

Policy documents reveal that NHIS transfers advance payments to HMOs on a quarterly basis.^17^ HMOs then make capitation payments and reimburse providers for fee-for-service claims. Capitation payments (N750/$4.41 per member per quarter) are paid to providers for a predetermined package of primary healthcare services. FFS payments are reimbursements for secondary care in the predetermined benefit package and providers are expected to make claims after services have been provided to enrolees. The payment levels are actuarially determined by NHIS and reimbursement rates are the same for public and private providers. HMOs are expected to make timely payments to healthcare facilities. Facilities are required to make fee-for-service claims monthly and these claims should be settled within 14 days of receipt by HMOs. HMOs are expected to make capitation and fee-for-service payments to providers on a monthly basis and send payment reports to the NHIS. In addition, HMOs are required to submit annual reports and audited accounts to NHIS.

Providers are not satisfied with the fee-for-service reimbursements; they report that payments do not cover costs of care, and that this negatively impacts on the quality of services offered to enrolees. Providers have a general reluctance to offer services that may potentially exceed the reimbursement ceiling, even if those services are judged to be in the best interests of enrolees.

*…*for instance, there was once an asthmatic patient came to the hospital, we had to nebulise; so we charged for nebulisation. An officer from Lagos, working with one of the HMOs came down, flew down to Enugu, to find out what is going on, that their doctor said that there is nothing like nebulisation [on the service list]. So, because it’s not listed, and you will not withhold that service because it is not listed. Our main focus is to save life, first of all.*’* (IDI with a private provider 4)

This level of discontent appears to be higher among private providers than public providers, who receive payment for government salaries in addition to capitation and fee-for-service payments for FSSHIP members.

Well, for a private individual, they may not break even; but for this hospital, because of the number of enrolees [in FSSHIP], that is the reason why we are breaking even. The capitation fee is not adequate, in my own opinion anyway, because if you consider a private institution, and maybe they may not have enough patients, they may not be able to meet up with the challenges; to give those drugs and other necessary services, but because this place is government owned, and we have reasonable number of patients; that is the reason why we are breaking even, we are trying to cover up. So it’s not that the capitation fee is enough but we have to manage. (IDI with a public provider 2)

Both public and private providers reported that they often experience delays in receiving capitation payments and reimbursements for fee-for-service claims from HMOs. The delay in fee-for-service reimbursement is partly due to a lengthy claim verification process. This process can take months due to the fact that any queries on claims by HMOs are documented and returned to providers for further clarification and then sent back to the HMOs. In addition, a small number of providers fail to send claim forms in a timely manner, affecting their own payments and the accounting processes of the relevant HMOs. The NHIS guidelines stipulate that the NHIS is required to supervise, monitor and perform regular audits of HMOs to ensure that healthcare providers are paid in a timely manner. Currently, the NHIS does not perform these tasks on a regular basis.

Delays in payment from HMOs, together with dissatisfaction with payment rates, have discouraged healthcare providers from treating FSSHIP members in a timely manner and with appropriate care; some beneficiaries have been refused treatment, because the providers are owed money by the HMO.

“…There is something they [providers] are doing now when you go to the hospital…, they will ask you to wait while they go to call the HMO to get approval to treat that illness… There was a day, I was there till evening, and I didn’t get the go ahead, and they asked me to go*…* (FGD with female FSSHIP member 8).

…NHIS patients are like the riffraff, poor people, the common masses, the nobodies. And so any time you come in there under platform of NHIS, they look down on you, you don’t get attention you require. (FGD with female FSSHIP member 2).

In addition to payment delays, providers report that payments rates have not been adjusted along with inflation rates since inception of the programme, and as a result, services to beneficiaries are constrained.

### Transparency in decision making

Under FSSHIP, NHIS is given authority to decide on benefit entitlements, provider payment rates and payment mechanisms and to select providers. Healthcare providers, particularly private providers, complain about the ‘top-down’ approach to decision making and their exclusion from the decision-making process, particularly relating to decisions on provider payment rates, and providers are of the opinion that NHIS does not clearly communicate the decisions that they have made.

NHIS convened an expert committee to define benefit packages. An actuarial study was undertaken to determine rates for capitation and fee for service. The expert committee was comprised of a diverse group of actors including representatives of HMOs, providers, the NHIS, civil society organisations, academia and the Federal Ministry of Health. Although a number of providers were part of the expert committee, there was discontent among providers about not being fully involved in the decision-making process on benefit packages and provider fees.

*…*we normally have agreement with them, though the agreement is just a verbatim of national health insurance agreement, the one that HMOs normally prepare. So, they are following NHIS formula. Most of the agreement, we don’t even question it because it is already prepared and handed over to us. So we are not participating in the preparation of the agreement. So it is just like unequal bargaining. The capitation fee, they just fixed it, we just swallow it as it is, whether it is enough or not enough. There is nothing we can say because it is a national thing*…*! (IDI with a public provider 2)

It’s terrible! It’s like playing a football match and somebody is bringing the referee and where the goal post will be. They will choose everything; chose their own tariff; but I think it’s changing now. Like personally, in our own hospital, if you are going to pay us fee-for-service, it must be my own tariff. You don’t bring a tariff and tell me to use; we must sit down, check the costing ourselves*….* (IDI with a private provider 5)

Providers are of the opinion that adequate and regular communication between NHIS, HMOs, providers and FSSHIP members is necessary and will foster a more satisfying relationship among all parties.

It’s going to benefit both the enrolees and the providers and even the HMOs because the thing is this: when we discuss, we are in the field, we see these patients, we see most of them that have these problems. Then if for example, an enrolee has a problem with a provider, and he reports to the HMOs, at least, the problem now, you now hear from the two sides; not just from the enrolee but from the provider. Now it’s going to be beneficial to everybody because there will be a kind of a feedback – getting an avenue to feedback from the field. So when you get feedback, you can evaluate. At least, it will be a source of evaluation for your own programme, for you to be able to say, okay, this thing, is it really working? What is the enrolee satisfaction? What is the provider satisfaction and all that? If the provider is not satisfied and the enrolee is not satisfied, obviously your programme is not working. (IDI with a private provider 5)

## Discussion

This article explores the relationship between purchasers (NHIS and HMOs) and healthcare providers in the FSSHIP, assessing its functioning from a strategic purchasing perspective and examining how policy levers such as accreditation, monitoring, provider payment mechanisms and decision-making mechanisms, all part of purchasing arrangements, have shaped this relationship.

The results of our analysis show that in practice, these policy levers are not being used in such a way as to produce the desired health service outcomes of efficiency and quality. For example, although the NHIS accredits healthcare providers to operate as part of FSSHIP, reaccreditation is rarely undertaken, and the quality of healthcare services delivered by FSSHIP registered providers is not monitored. Many HMOs regularly undertake financial audits of healthcare providers to ensure claims for fee-for-service payments are justified, but similar, rigorous monitoring of clinical quality is infrequent.

The study identified poor governance in the two-tiered purchasing mechanism and found it to be a major cause of poor implementation of strategic purchasing tools. A combination of financial and human resource constraints and a range of political issues have resulted in the NHIS being ineffective in both providing stewardship to HMOs and monitoring the performance of HMOs in their role as purchasing administrators. In addition, NHIS has failed to provide effective direction to HMOs on their expected tasks, and HMOs lack clarity on what aspects of healthcare provider performance they are supposed to monitor and how this should be done. This has resulted in wide variation in how HMOs undertake their FSSHIP purchasing administrator roles. Ambiguous policy design and limited capacity of purchasers constrain the effective use of strategic purchasing levers by purchasers. These findings were also identified as challenges to strategic purchasing in some other LMIC settings.^2 11 26 27^

Other countries that employ private firms to act as purchasing administrators in public mandatory health financing mechanisms experience similar problems to those of the HMOs under the Nigerian FSSHIP. For example, in India, the Rashtriya Swasthya Bima Yojana (RSBY) uses insurance companies to act as purchasing administrators, and the insurance companies are responsible for enrolling members, accrediting hospitals, processing claims and reimbursing hospitals. However, there is no clear measure in place for the Indian government to monitor the performance of the private firms and, in practice, low-income beneficiaries are still making out-of-pocket payments when accessing healthcare services.^10^ The Georgian government also contracts private firms to manage the country’s public insurance scheme for the poor population. However, unlike the FSSHIP, beneficiaries have an annual opportunity to change their insurance administrator if they are unsatisfied with services provided, potentially motivating insurance administrators to deliver quality services. Despite this opportunity, evidence reveals that improper practices by private insurance companies, including delayed enrolment and overly aggressive utilisation management, are critical factors in preventing the desired impact of the scheme from being realised and so unable to protect the poor from catastrophic healthcare expenditure.^28^

Healthcare providers often experience delayed payment from HMOs and have expressed strong dissatisfaction with payment rates. While payment mechanisms can be used to encourage healthcare providers to supply quality and efficient healthcare services, delays in payments and dissatisfaction with payment rates have had an adverse effect on healthcare service delivery under FSSHIP. Private providers appear to be more affected by payment delays and perceived low payment rates than public providers.^29 30^ Similar issues associated with the timing and rates of payments have also been reported in India and Malawi.^10 27^ In Thailand, where payments are adjusted annually in line with inflation rates, less provider dissatisfaction is reported.^11 31^

Two factors appear to be at play. First, current policy guidelines give NHIS absolute autonomy and consequently, the decision-making process has not been inclusive of providers after the initial consultations at the programme inception. Second, an absence of clear communication and feedback channels further constrains transparency in decision making by NHIS. Decisions are perceived to be top-down, and this has led to dissatisfaction among providers who are already restricted from exercising the freedom to choose HMOs. Providers want to be involved in deciding benefit packages, capitation rates and rates of fee-for-service reimbursements. In another Nigerian state, the failure to include providers in the FSSHIP decision-making process caused providers to be disinterested in the programme and led to the state choosing not to adopt the FSSHIP.^26^

The experiences and opinions of FSSHIP held by different providers are affected by the time at which they entered the programme, with providers who were present at the inception of the programme feeling that they were included in some aspects of the decision making and providers who joined later feeling less included. Failure of NHIS to maintain communication with providers has meant that later entrants into FSSHIP are not well engaged with NHIS. Many providers are of the opinion that purchasing decisions are made by NHIS with a top-down approach. As a result, providers tend to be uncommitted to the ideals of the programme, especially when decisions do not align with their own interests. Poor communication and hierarchical decision making have been widely identified as constraints.^2^ Vested interests by influential and powerful people in the NHIS organisation and government may be an additional constraint.^32^

The case study approach of the study may limit the generalisability of the study findings to the rest of the country. Further studies are required to obtain robust theoretical inferences and/or identify contrasts between states to further understand the strategic purchasing relationship between the two tiers of purchasers and the healthcare providers operating under FSSHIP. It is also important to look at the purchaser–provider relationship in association with other purchasing relationships and examine the role that citizens and government can play in the governance of the purchaser–provider relationship.

The failure to use purchasing strategically has potentially negative consequences for quality and efficiency in healthcare service provision. Delays in payments to healthcare providers, coupled with dissatisfaction with provider payment rates appear to have resulted in healthcare providers having unfavourable attitudes towards FSSHIP member patients. The FSSHIP scheme currently suffers from low participation rates.^33^ The fact that FSSHIP members have negative experiences when accessing healthcare services may further exacerbate problems with the uptake of the FSSHIP scheme by formal sector workers. Furthermore, low coverage of the population constrains the ability of purchasers to use economic power to undertake strategic purchasing. The experience of FSSHIP in Nigeria is illustrative of the vicious cycle caused when a purchasing mechanism malfunctions. Beneficiaries in the Indian RSBY have also experienced suboptimal care due to providers’ dissatisfaction with payments.

In conclusion, FSSHIP purchasers are not using strategic purchasing tools to improve healthcare service provision for members. This is due to mal-functioning of the two-tiered purchasing mechanism wherein NHIS does not effectively provide stewardship in monitoring and guiding HMOs to fulfil their roles and responsibilities as purchasing administrators. With accreditation left to NHIS, they tend to overlook failures as they are interested in extending healthcare coverage and healthcare spending. The lack of HMO choice by enrolees, as well as the conflict of interest in the participation of HMOs as NHIS governors are also sources of failure.

A number of policy recommendations flow from these findings. First, the governance framework for healthcare purchasing in the NHIS should be revised to ensure clarity in the roles of purchasers (NHIS and HMOs) at both levels in of the system. Second, NHIS requires capacity building for active supervision, monitoring and evaluation of health services provided to enable it provide stronger stewardship of the FSSHIP. Third, the use of an independent accreditation agency would reduce potential conflicts and improve transparency in choice of HMOs. HMOs as part of the governing body of NHIS needs to be reviewed as this is also a source of conflict.
